# Acute kidney injury and chronic kidney disease in Chile: Temporal trends in hospitalization rates from 2010 to 2019

**DOI:** 10.1371/journal.pone.0337640

**Published:** 2025-12-11

**Authors:** Luis Celis, Jenny Ruedlinger, Cinthya Leiva, Camilo G. Sotomayor, Roberto Jalil, Sandra Cortés

**Affiliations:** 1 School of Public Health, Pontificia Universidad Católica de Chile, Santiago, Chile; 2 Advanced Center for Chronic Diseases (ACCDiS), Pontificia Universidad Católica de Chile, Santiago, Chile; 3 Radiology Department, Clinical Hospital University of Chile, University of Chile, Santiago, Chile; 4 Laboratory of Medical Informatics and Telemedicine, Institute of Biomedical Sciences, Faculty of Medicine, University of Chile, Santiago, Chile; 5 Department of Nephrology, Pontificia Universidad Católica de Chile, Santiago, Chile; 6 Centre for Sustainable Urban Development (CEDEUS), Pontificia Universidad Católica de Chile, Santiago, Chile; Universita degli Studi della Campania Luigi Vanvitelli Scuola di Medicina e Chirurgia, ITALY

## Abstract

**Background:**

Kidney diseases pose considerable challenges to health systems affecting millions of people worldwide. In Latin America there are limited epidemiological studies on these conditions.

**Objective:**

This study aimed to describe the national age-adjusted hospitalization trends for acute kidney injury (AKI) and chronic kidney disease (CKD) in the Chilean population.

**Methods:**

We conducted a retrospective study using a secondary data from hospitalized cases diagnosed with AKI and CKD in Chile, from 2010 to 2019. Age-adjusted hospitalization rates and JoinPoint regression analysis were performed.

**Results:**

Between 2010 and 2019, 26,715 hospitalizations of patients diagnosed with AKI and 99,816 patients with CKD were recorded. Nationally, long-term trends in age-adjusted hospitalization rates for AKI increased by 9.1% annually (95% CI: 8.0 to 10.5), whereas CKD hospitalization rates declined by 4.35% (95% CI: −5.76 to −2.99) over the study period. Short-term trends in AKI hospitalization rates showed initial declines in Maule between 2010–2017 (−7.97%, 95% CI: −13.63 to −6.04) followed by increases from 2017 to 2019 (8.45%, 95% CI: −4.08 to 17.66), whereas Valparaíso (7.2%, 95% CI: 5.2 to 9.5) experienced steady growth. Although CKD rates generally declined, central regions such as O’Higgins and Maule showed increases between 2016–2019 (10.6%, 95% CI: 5.1 to 19.9) and 2017–2019 (8.5%, 95% CI: −4.1 to 17.7), respectively.

**Conclusion/Interpretation:**

This study highlights AKI as a growing public health challenge nationwide, with increasing hospitalization rates. At the same time, CKD hospitalizations show a reduction, although regional disparities remain a concern. Efforts should focus on enhanced integral surveillance to address these trends effectively across the country.

## Introduction

Kidney diseases have become the 10th leading cause of death globally, with mortality increasing from 813,000 in 2000 to 1.3 million in 2019 [1]. This growing burden presents considerable challenges to health systems worldwide, not only leading to high mortality rates, but also impacting quality of life and healthcare costs [2]

Acute kidney injury (AKI) is defined as a sudden decrease in kidney function, usually occurring over hours to days, often caused by conditions such as sepsis, dehydration, or nephrotoxic medications, among others [3]. AKI is associated with high morbidity and mortality rates and serves as a risk factor for developing chronic kidney disease (CKD) [3]. Globally, AKI is estimated to affect more than 13 million people annually, with incidence rates varying widely among hospitalized patients, exceeding 50% in those in intensive care units (ICU), and also contributing to approximately 1.7 million deaths per year [4,5]. In Latin America, AKI present a high burden, particularly in critically ill patients, with incidence rates varying across regions and healthcare settings [6]. Recent evidence from Brazil reported an incidence of 53.3% and a 7-day prevalence of 72.5% among ICU patients [7], while a systematic review in Mexico estimated a pooled prevalence of 35% across hospitalized populations [8].

CKD is characterized by abnormalities in kidney structure or function, present for more than 3 months with a reduced glomerular filtration rate (GFR) of less than 60 mL/min/1.73 m². These abnormalities include markers of kidney damage, such as albuminuria, urinary sediment abnormalities, electrolyte and other abnormalities due to tubular disorders, abnormalities detected by histology, structural abnormalities detected by imaging, or a history of kidney transplant [9]. The global prevalence of CKD is estimated to be around 10%, affecting more than 800 million people [10]. In Latin America, CKD prevalence varies widely, with a regional median of 10.2% (95% CI: 8.4–12.3%) and an estimated prevalence of 10.2% (95% CI: 9.5–10.8) in Chile [11]. Additionally, data from Chile’s National Health Survey (ENS) 2016–2017 indicate that 3.2% (95% CI: 2.4–3.8%) of adults aged 18 years and older exhibited reduced kidney function, while 15.4% of those over 40 years old had CKD (stages 1–5) [12] Beyond Chile, community-based studies reveal important heterogeneity across the region: in Nicaragua, adults aged 15–59 years demonstrated an overall CKD prevalence of 8.6% [13] among Truká Indigenous adults in Cabrobó, Brazil, prevalence was 10% (95% CI, 8.6–11.5%) [14]; whereas in the Tumbes region of Northern Peru, a population-based survey reported a markedly lower prevalence of reduced eGFR (<60 mL/min/1.73 m²) at 1.7% (95% CI, 1.1–2.5%) [15].

Both AKI and CKD present a complex interplay of traditional and non-traditional risk factors, which emphasize the multifaceted nature of these diseases. In Latin America, various risk factors and social determinants, such as socioeconomic disparities, limited access to health care, and diverse environmental factors, likely contribute to the growing burden of kidney disease [16–18]. Despite this, kidney diseases have not received the attention they deserve on the global, regional or Chilean public health agenda [18–20]. The international consensus on the need to prioritize chronic kidney disease in public health policy underscores this gap [21].

Therefore, monitoring and understanding hospitalization trends for patients with conditions such as AKI and CKD are essential, not only to evaluate current health systems and resource allocation, but also as a valuable source of information in future health policies. The results of these data can contribute to improve resource allocation or the development of specific interventions [22,23], so it could be used to guide health policies aimed at reducing the burden of these conditions in the Chilean population.

This study aims to describe national estimates of age-adjusted hospitalization trends for both AKI and CKD in the Chilean population. Given the significant global and national burden of these conditions and the absence of recent national data on kidney disease in Chile, our research addresses a critical knowledge gap.

## Methods

### Ethic statement

The Scientific Ethics Committee for Health Sciences at the Pontificia Universidad Católica de Chile reviewed and approved the study protocol (Resolution Act: protocol ID 241004004 of October 10, 2024).

### Study design

A retrospective ecological study was conducted nationwide based on secondary data sources from hospitalized cases diagnosed with AKI and CKD during the last decade available, from 2010 to 2019.

### Data sources

This database contains hospitalizations from the public and private health care system where diagnoses were coded under the International Classification of Diseases (ICD-10) (available at https://deis.minsal.cl/ #datosabiertos). The data were accessed for research purposes between September 2 and September 30, 2023. At no point during or after data collection did the authors have access to any information that could identify individual participants. The database includes demographic data, dates of admission and discharge, primary and secondary diagnoses, region of residence, the length of stay (sum of days hospitalized), and in-hospital mortality (which refers to whether the patient died or not). Only hospitalizations with a principal diagnosis of AKI and CKD, using the ICD-10 codes N17 and N18, respectively, were included.

### Data analysis

To describe demographic variables, proportions were used for categorical variables and mean and standard deviation or median and range for continuous variables. Differences in proportions were assessed using the Chi-squared test, while the Student’s t-test and the Wilcoxon rank-sum test were used for normally and not normally distributed continuous variables, respectively. Age-adjusted hospitalization rates were calculated through direct standardization method. The standard population used was the World Health Organization (WHO) World Standard Population [24] and the national population data were sourced from the National Institute of Statistics (INE; Instituto Nacional de Estadísticas) [25]. All analyses were conducted using R version 4.3.3. Statistical significance was set at a p-value <0.05.

Using the average age-adjusted rates by region from 2010 to 2019, regional maps were created using ArcMap 10.5 software (ArcGIS). Administrative boundary shapefiles were obtained from the Biblioteca del Congreso Nacional de Chile (BCN; https://www.bcn.cl/siit/mapas_vectoriales). All maps are original creations by the authors and suitable for publication under the CC BY 4.0 license.

The Joinpoint Regression Program version 5.2.0 was used to estimate the Average Annual Percent Change (AAPC) and the Annual Percent Change (APC) in age-adjusted hospitalization rates. The AAPC provides a summary measure of the overall trend, while the APC represents the rate of change per year over a specified period. Both AAPC and APC were calculated with 95% confidence intervals (CI) to assess statistical significance.

Joinpoint regression utility resides in that it identifies the year(s) when a trend change occurs by connecting several different line segments on a log scale at ‘joinpoints’. This method begins by fitting a straight line (zero joinpoints) and sequentially tests whether introducing additional joinpoints significantly improves the model. Each joinpoint represents a year in which a statistically significant change in the slope of the trend occurred. The final model therefore consists of a series of connected linear segments on a logarithmic scale, with each “joinpoint” marking a significant shift in the trajectory of the rates [26].

## Results

### AKI and CKD hospitalizations

In Chile, between 2010 and 2019, a total of 26,715 hospitalizations of patients with AKI and 99,816 of patients with CKD were recorded (Table 1). Hospitalizations were slightly more frequent in men, both in AKI (53.9%) and CKD (52.7%). The average age of hospitalized patients with AKI was 64.8 years (± 20.1), being slightly lower in men (64.0 ± 19.7 years) than in women (65.8 ± 20.5 years). with a significant difference (p < 0.001). The average age of CKD patients was 60.1 years (± 18.7), with no significant differences between men and women (p = 0.98).

**Table 1:** Characteristics of patients hospitalized with acute kidney injury (AKI) and chronic kidney disease (CKD) in Chile from 2010 to 2019.

Variable	Total	Male	Female	p-value
**ICD-10 N17 codes for AKI**				
**Number of hospitalizations** (%)	26715 (100)	14396 (53.9)	12319 (46.1)	
Age (mean, SD)	64.8 (± 20.1)	64.0 (± 19.7)	65.8 (± 20.5)	< 0.001
**Age group (%)**				
0–19	1065 (4.0)	521 (3.6)	544 (4.4)	< 0.001
20–39	2308 (8.6)	1339 (9.3)	969 (7.9)	
40–59	4918 (18.4)	2799 (19.4)	2119 (17.2)	
60–79	11789 (44.1)	6576 (45.7)	5213 (42.3)	
80+	6635 (24.8)	3161 (22.0)	3474 (28.2)	
Length of stay (days) (median. range)	6 (1 - 438)	6 (1 - 438)	6(1 - 387)	< 0.001
**In-hospital Mortality** (%)				
Dead	2501 (9.4)	1251 (8.7)	1250 (10.1)	< 0.001
Alive	24214 (90.6)	13145 (91.3)	11069 (89.9)	
**ICD-10 N18 codes for CKD**				
**Number of hospitalizations** (%)	99816 (100)	52583 (52.7)	47233 (47.3)	
Age (mean, SD)	60.1 (± 18.7)	60.2 (± 18.5)	60.1 (± 19.0)	0.978
**Age group (%)**				
0–19	4257 (4.3)	2220 (4.22)	2037 (4.31)	< 0.001
20–39	9608 (9.6)	4781 (9.09)	4827 (10.2)	
40–59	27111 (27.2)	14431 (27.4)	12680 (26.8)	
60–79	46056 (46.1)	24848 (47.3)	21208 (44.9)	
80+	12784 (12.8)	6303 (12.0)	6481 (13.7)	
Length of stay (days) (median. range)	6 (1 - 946)	6 (1 - 946)	5 (1 - 806)	< 0.001
**In-hospital Mortality**				
Dead	5725 (5.7)	2976 (5.7)	2749 (5.8)	0.282
Alive	94091 (94.3)	49607 (94.3)	44484 (94.2)	

Source: Data from the vital statistics system, hospital discharges, DEIS, MINSAL.

When classified by age group, in both AKI and CKD, men presented higher proportions in the 40- to 79-year-old groups, while women predominated in the 80-year-old or older group (13.7% versus 12.0% in men, p < 0.001). Overall, in-hospital mortality was 9.4% for AKI and was 5.7% for CKD. Total length of stay during the study period ranged from 1 to 438 days for AKI and 1–946 days for CKD, with a median of 6 days for both.

The highest proportion of hospitalizations of patients with AKI and CKD were for unspecified acute kidney injury (N17.9) and unspecified CKD (N18.9), representing 89.7% and 70.1% of hospitalizations, respectively (S1 Table 1 and S1 Table 2 in S1 File).

As shown in Table 2, the age-adjusted hospitalization rate (standardized to the WHO World Standard Population) of patients diagnosed with AKI exhibit an increasing trend, rising from 7.7 to 17.0 per 100,000 inhabitants between 2010 and 2019, while those of patients diagnosed with CKD display a decreasing trend, declining from 54.1 to 37.1 per 100,000 inhabitants.

**Table 2:** Age-adjusted hospitalizations rates for acute kidney injury (AKI) and chronic kidney disease (CKD) in Chile from 2010 to 2019.

Year	Number of AKI Hospitalizations	Number of CKD Hospitalizations	AKI age adjusted rates	CKD age adjusted rates
2010	1,491	10,198	7.7	54.1
2011	1,741	10,172	8.7	52.5
2012	1,941	11,474	9.4	58.2
2013	2,177	9,712	10.3	47.5
2014	2,166	9,176	9.9	43.7
2015	2,665	9,709	11.9	45.1
2016	3,050	9,538	13.3	43.1
2017	3,445	9,585	14.5	42.2
2018	3,766	9,273	15.5	39.6
2019	4,273	8,964	17.0	37.1
2010 - 2014	9,516	50,732	9.2	51.2
2015 - 2019	17,199	47,069	14.4	41.4
2010 - 2019	26,715	97,801	11.8	46.3

Rates per 100,000 inhabitants. WHO World Standard Population. Source: Data from the vital statistics system, hospital discharges, DEIS, MINSAL.

**Fig 1:**
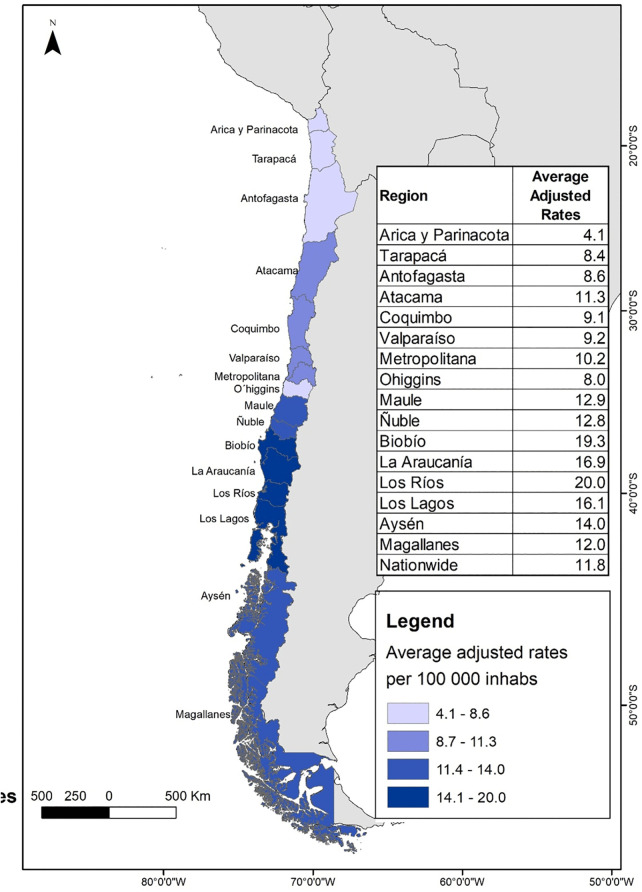
Average age-adjusted hospitalization rates (standardized to the WHO World Standard Population) for patients with acute kidney injury (AKI) by region in Chile, 2010-2019. Map created by the authors using open-access shapefiles from the Biblioteca del Congreso Nacional de Chile (BCN) and population data from the Instituto Nacional de Estadísticas (INE).

**Fig 2:**
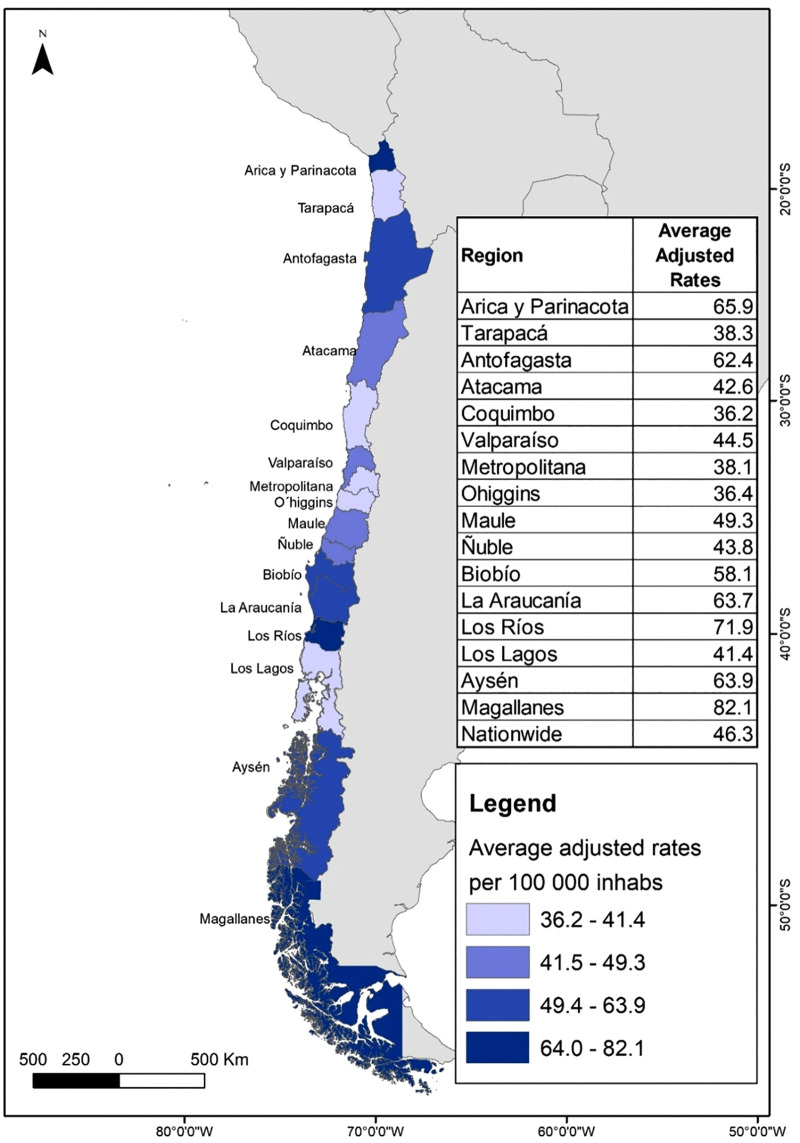
Average age-adjusted hospitalization rates (standardized to the WHO World Standard Population) for patients with chronic kidney disease (CKD) by region in Chile, 2010-2019. Map created by the authors using open-access shapefiles from the Biblioteca del Congreso Nacional de Chile (BCN) and population data from the Instituto Nacional de Estadísticas (INE).

In the northern regions of Chile, the average age-adjusted hospitalization rates (WHO standard population) during the period shows that rates of patients diagnosed with AKI range from 4.1 per 100.000 inhabitants in Arica and Parinacota to 11.3 per 100.000 inhabitants in Atacama (Fig 1). For CKD, rates range from 38.3 per 100,000 inhabitants in Tarapacá to 65.9 per 100,000 inhabitants in Arica and Parinacota (Fig 2).

In central regions, AKI age-adjusted hospitalization rates (WHO standard population) are relatively homogeneous, ranging between 9.1 in Coquimbo and 10.2 in the Metropolitan Region. In this same area, CKD rates show more variability, with Coquimbo reaching 36.2 and Valparaíso with a higher rate of 44.5.

In the central-southern regions for AKI, Bío-Bío have the highest age-adjusted hospitalization rate (19.3) and O’Higgins the lowest (8.0). For CKD, the rates are more consistent, although Bío-Bío (58.1) and Maule (49.3) stand out with relatively high rates.

In southern regions we also find high age-adjusted hospitalization rates for AKI, e.g., Los Ríos with 20.0 per 100.000 inhabitants, meanwhile the rates for CKD are even higher, with La Araucanía (63.7) and Los Ríos (71.9) standing out.

In austral regions, AKI rates vary between 12.0 in Magallanes and 14.0 in Aysén, while CKD presents the highest figures in the country, i.e., Magallanes with 82.1 and Aysén with 63.9.

### National and regional trends in hospitalizations rates

To summarize the long-term trends in age-adjusted hospitalization rates (WHO standard population) for both conditions we used the average annual percent change (AAPC) which provides a single measure of the overall rate of change across a study period, smoothing out year-to-year variability.

At the national level, the age-adjusted hospitalization rates among patients diagnosed with AKI increased by 9.1% annually (95% CI: 8.0 to 10.5), while CKD hospitalization rates declined by 4.35% (95% CI: −5.76 to −2.99) over the study period (specific data are available at S1 Table 3 and S1 Table 4 in S1 File).

**Fig 3:**
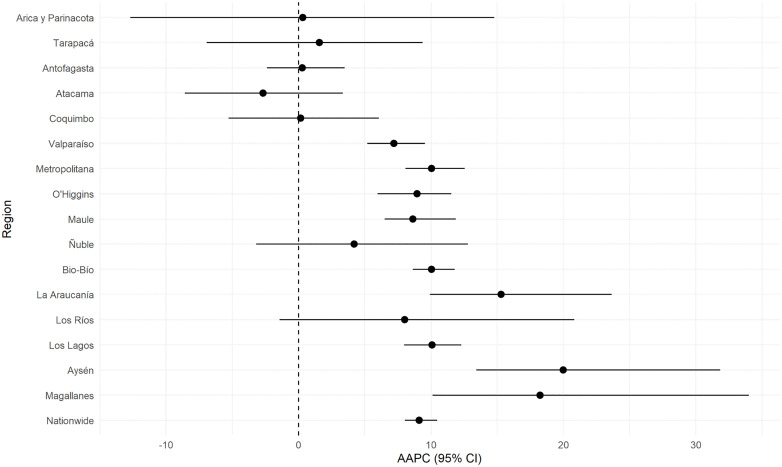
Regional variations in the average annual percent change (AAPC) of AKI age-adjusted hospitalization rates (standardized to the WHO World Standard Population) in Chile from 2010 to 2019.

At regional level, for AKI (Fig 3), the most pronounced increases were observed in southern and austral regions, such as Aysén (+20.0%, 95% CI: 13.4 to 31.8), Magallanes (+18.2%, 95% CI: 10.1 to 34.0), and La Araucanía (+15.3%, 95% CI: 9.9 to 23.6). Regions such as Metropolitan, Bio-Bío, and Los Lagos also exhibited moderate increases of approximately +10%, while the Atacama region was the only region that showed a non-statistically significant declined (−2.7%, 95% CI: −8.6 to 3.3).

**Fig 4:**
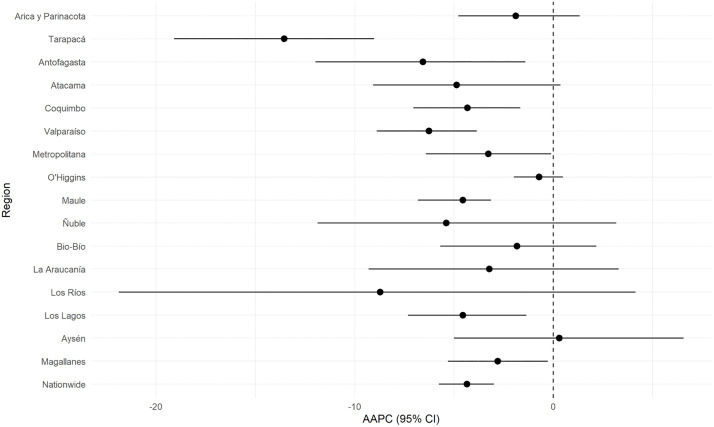
Regional variations in the average annual percent change (AAPC) of CKD age-adjusted hospitalization rates (standardized to the WHO World Standard Population) in Chile from 2010 to 2019.

In contrast, the AAPC for CKD age-adjusted hospitalization rates (Fig 4) exhibited predominantly decreasing trends, with the most pronounced declines observed in Tarapacá (−13.5%, 95% CI: −19.1 to −9.0), Antofagasta (−6.6%, 95% CI: −12.0 to −1.4), and Valparaíso (−6.3%, 95% CI: −8.9 to −3.9). Moderate reductions were observed in Coquimbo, Maule, and Metropolitan Region, with AAPCs ranging between −3.3% and −4.6%.

To complement the AAPC, the annual percent change (APC) was calculated to assess year-to-year variations in age-adjusted hospitalization rates (WHO standard population). This approach allows for a more detailed view of short-term trends and shifts.

**Fig 5:**
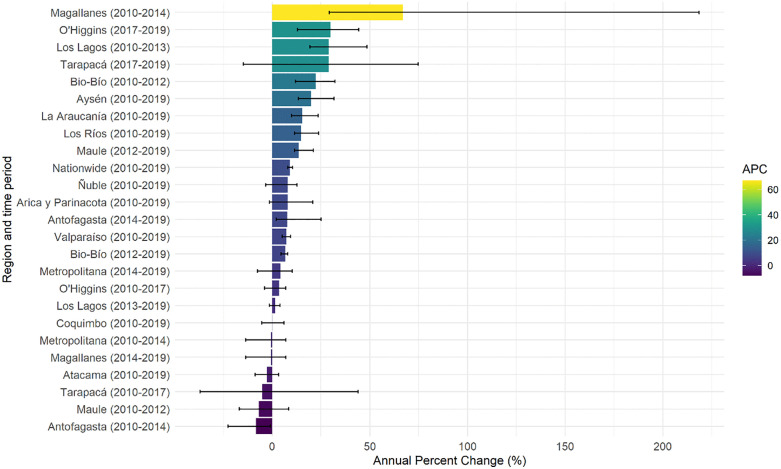
Annual Percent Change (APC) in age-adjusted hospitalization rates (standardized to the WHO World Standard Population) for AKI by region in Chile, 2010-2019. The figure illustrates regional variations in hospitalization trends with fluctuations observed across years. The APC are divided into four ranges and differentiated by colour.

The APC for AKI (Fig 5), the Maule region shows a decrease between the years 2010–2017 (−7.97%, 95% CI: −13, 63 to −6.04), followed by an increase in the period 2017–2019 (8.45%, 95% CI: −4.08 to 17.66). Similar trends were observed in regions such as Antofagasta and O’Higgins, that showed contrasting trends in different periods, with initial drops followed by subsequent increases. On the contrary, regions such as Valparaíso (7.2%, 95% CI: 5.2 to 9.5) and Bío-Bío (6.8%, 95% CI: 4.6 to 8.0) showed sustained increases throughout the entire time-period.

**Fig 6:**
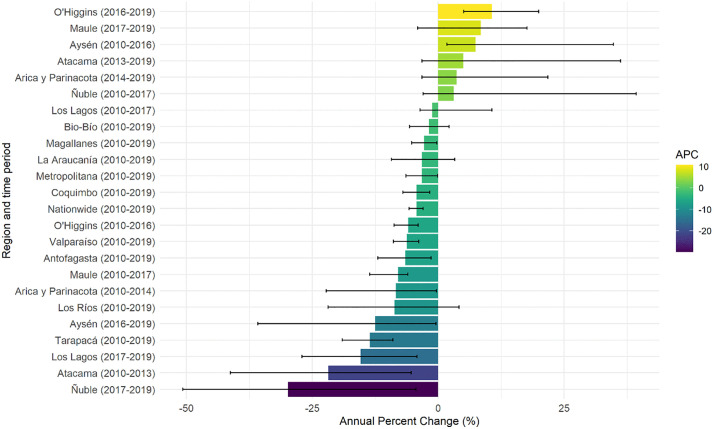
Annual Percent Change (APC) in age-adjusted hospitalization rates (WHO standard population) for CKD in Chile, 2010 - 2019. The figure illustrates regional variations in hospitalization trends with fluctuations observed across years. The APC are divided into four ranges and differentiated by colour.

On the other hand, for CKD (Fig 6), most regions experienced a decrease in age-adjusted hospitalization rates, with significant reductions in Tarapacá (−13.55%, 95% CI: −19.09 to −9.01) and Antofagasta (−6.56%, 95% CI: −11.97 to −1.40). Some regions, such as O’Higgins and Maule, showed mixed trends, with initial decreases followed by increases in subsequent years.

## Discussion

To the best of our knowledge, this is the first study that describes trends in hospitalizations for AKI and CKD in Chile, revealing trends in hospitalization rates between 2010 and 2019. Generally, epidemiological data on AKI and CKD in Latin America are scarce and vary between countries, where most studies are based on hospital settings or specific population groups, and the scarcity of national studies limits knowledge about the prevalence and burden of these diseases in the region [27,28].

Our results show that in Chile, the age-adjusted rates of AKI more than doubled over the decade. This increase is consistent with reports from other Latin American countries [4]. For instance, in Peru the age-standardized incidence rate of AKI per 100,000 people increased by 15.2% [29]. A comparable trend also has been observed in the United States, a developed country in the Americas, where the hospitalization rate (95% CI) increased from 23.1 (21.5 to 24.8) in the year 2000 to 55.3 (54.1 to 56.6) in 2014 [30]. These patterns emphasize the growing burden of AKI on the healthcare system globally [31]. For CKD, while our findings show an overall downward trend in age-adjusted hospitalization rates, although modest, this decrease may suggest potential improvements in disease management and prevention strategies [32,33], this contrasts with global and regional trends where CKD continues to rise [2,34,35].

Our study indicates that both conditions are slightly more frequent in men, which contrasts with previous reports that CKD is more prevalent in women [36], this sex differences also vary across countries and regions and illustrate the complexities of CKD epidemiology [36,37]. For instance, in Nicaragua CKD prevalence was higher among men (12.5%) than women (6.1%) [13], whereas among Truká Indigenous adults in Brazil, prevalence was higher in women (12.4%) than men (6.9%), reaching 35.7% in women aged ≥60 years [14]. Evidence from the Hispanic Community Health Study in the United States further demonstrates this heterogeneity, with CKD prevalence ranging from 7.4% in South American women to 16.6% in Puerto Rican women, and from 11.2% in South American men to 14.8% overall [38]. On the other hand, the highest number of hospitalizations are observed in older age groups, aligning with global and regional trends associating these conditions with aging populations [39,40], suggesting a complex interplay of factors such as comorbidities, gender disparities, severity of illness, and possibly heterogeneous levels of healthcare access and quality among the elderly [41,42].

Regional variations in hospitalization rates for AKI and CKD are evident, reflecting local epidemiological dynamics. These may be caused by differences in healthcare infrastructure, socioeconomic factors, environmental exposures, and local variations in risk factors [43–46]. Moreover, issues of inequality and limited access to healthcare are persistent challenges in Latin America and Chile [47–49].

Thus, the rising trend in AKI hospitalizations, and CKD in some regions of our country, underscores the need for targeted interventions to address this issue, particularly in high-risk regions and populations, i.e., regions with high prevalence of diabetes mellitus or hypertension. Strategies could include improving early detection and management, enhancing public and healthcare professional awareness, and addressing modifiable risk factors such as hypertension and diabetes [50–52].

### Limitations

Limitations of our study include reliance on hospitalization and hospital discharge data, which does not guarantee that AKI or CKD were the direct cause of the hospitalizations, but rather conditions present in the patients, furthermore it can overestimate AKI and CKD prevalence in the general population since there may be one or more hospitalizations/discharges of the same patient, and in this study the condition of re-entry into a medical facility for the same pathology was not measured. Additionally, as this is an ecological study based on aggregated data, it is subject to the ecological fallacy—associations observed at the population level may not hold at the individual level, and causal inferences about individual risk factors cannot be made. Also, the possibility of misclassification due to the use of ICD-10 codes cannot be ruled out. This lack of clinical detail prevents proper interpretation because it obscures differences in etiology, severity, and contributing factors. However, the use of administrative codes for case identification is a common approach in epidemiological research and ensures comparability with other time-series studies based on hospital discharge records. Future research efforts should consider integrating outpatient data and longitudinal studies to provide a more comprehensive assessment of AKI and CKD outcomes and burden in Chilean and Latin American population [53,54].

## Conclusion

Our study contributes to the current understanding of kidney health in Chile. The findings underline the growing impact of AKI on public health, characterized by increasing hospitalization rates and significant regional disparities, while the data also suggest some positive trends in reducing CKD hospitalizations. Notably, the vast majority of hospitalizations for both AKI and CKD were coded as unspecified (N17.9 and N18.9, respectively), representing 89.7% and 70.1% of cases. This finding underscores the need to further explore the underlying causes and associated risk factors, in order to improve diagnostic precision and inform targeted prevention strategies. Addressing these challenges requires comprehensive public health strategies to assess regional disparities, enhance disease surveillance strategies, and implement targeted interventions to mitigate the public health impact. Importantly, incorporating AKI and CKD into national chronic disease surveillance frameworks would strengthen the capacity of policymakers to monitor trends, allocate resources more efficiently, and design effective prevention and management programs. Furthermore, this integration would allow for targeted preventive actions at the local level to address regional disparities. Future research should focus on identifying the specific drivers behind the trends observed and evaluating the long-term impact of public health interventions on AKI and CKD outcomes in Chile.

## Supporting information

S1 File Supplementary tables containing detailed summaries of AKI and CKD hospitalizations and regional trends in Chile from 2010 to 2019. (DOCX)
