# Supplementary material for: Acute kidney injury and chronic kidney disease in Chile: Temporal trends in hospitalization rates from 2010 to 2019
Source: PLoS One. 2025 Dec 11;20(12):e0337640. doi: 10.1371/journal.pone.0337640 (PMC12697955; doi:10.1371/journal.pone.0337640)
Supplement: S1 File — (DOCX) [file pone.0337640.s001.docx]

**Supplementary materials for:**

**Acute Kidney Injury and Chronic Kidney Disease in Chile: Temporal Trends in Hospitalization Rates from 2010 to 2019.**

**Table of Contents**

| Item | Content | Page |
| --- | --- | --- |
| 1 | S1 Table 1. Summary of hospitalizations for acute kidney injury (AKI) by ICD code in Chile from 2010 to 2019. | 2 |
| 2 | S1 Table 2. Summary of hospitalizations for chronic kidney disease (CKD) by ICD code in Chile from 2010 to 2019. | 3 |
| 3 | S1 Table 3. Annual Percent Change (APC) in Age-Adjusted Hospitalization Rates for Acute Kidney Injury by Region in Chile from 2010 to 2019. | 4 |
| 4 | S1 Table 4. Annual Percent Change (APC) in Age-Adjusted Hospitalization Rates for Chronic Kidney Disease, by Region in Chile from 2010 to 2019. | 6 |

| **Supplementary Table 1.** Summary of hospitalizations for acute kidney injury (AKI) by ICD code in Chile from 2010 to 2019. | | | | | | |  |
| --- | --- | --- | --- | --- | --- | --- | --- |
|  |  |  |  |  |  |  |  |
| **Variable** | **ICD-10 N17 codes for AKI** | | | | | |  |
|  | **Total** | **N17.0** | **N17.1** | **N17.2** | **N17.8** | **N17.9** |  |
| **Number of hospitalizations** (%) | 26,715 (100) | 787 (2.9) | 63 (0.2) | 152 (0.6) | 1,741 (6.5) | 23,972 (89.7) |  |
| **Sex (%)** |  |  |  |  |  |  |  |
| Men | 14,396 (53.9) | 422 (53.6) | 40 (63.5) | 83 (54.6) | 970 (55.7) | 12,881 (53.7) |  |
| Women | 12,319 (46.1) | 365 (46.4) | 23 (36.5) | 69 (45.4) | 771 (44.3) | 11,091 (46.3) |  |
| **Age (mean, SD)** | 64.8 (± 20.1) | 61.5 (± 21.3) | 60.8 (± 21.0) | 65.4 (± 19.4) | 62.2 (± 21.6) | 65.2 (± 19.9) |  |
| **Age group (%)** |  |  |  |  |  |  |  |
| 0 – 19 | 959 (3.6) | 33 (4.2) | 2 (3.2) | 2 (1.3) | 86 (4.9) | 836 (3.5) |  |
| 20 – 39 | 2,274 (8.5) | 101 (12.8) | 9 (14.3) | 16 (10.5) | 215 (12.4) | 1,933 (8.1) |  |
| 40 – 59 | 4,697 (17.6) | 155 (19.7) | 12 (19) | 26 (17.1) | 285 (16.4) | 4,219 (17.6) |  |
| 60 – 79 | 11,487 (43.0) | 317 (40.3) | 29 (46) | 65 (42.8) | 732 (42) | 10,344 (43.1) |  |
| 80+ | 7,298 (27.3) | 181 (23) | 11 (17.5) | 43 (28.3) | 423 (24.3) | 6,640 (27.7) |  |
| Length of stay (days) | 6 (1 - 438) | 7 (1 - 100) | 5 (1 - 83) | 7 (1 - 123) | 6 (1 - 194) | 6 (1 - 438) |  |
| (median. range) |  |  |  |  |  |  |  |
| **In-hospital Mortality** (%) |  |  |  |  |  |  |  |
| Dead | 2,501 (9.4) | 56 (7.1) | 10 (15.9) | 11 (7.2) | 123 (7.1) | 2301 (9.6) |  |
| Alive | 24,214 (90.6) | 731 (92.9) | 53 (84.1) | 141 (92.8) | 1,618 (92.9) | 21,671 (90.4) |  |
| **ICD-10 codes:** **N17.0** Acute kidney failure with tubular necrosis. **N17.1** Acute kidney failure with acute cortical necrosis. **N17.2** Acute kidney failure with medullary necrosis. **N17.8** Other acute kidney failure. **N17.9** Acute kidney failure, unspecified | | | | | | |  |

| **Supplementary Table 2.** Summary of hospitalizations for chronic kidney disease (CKD) by ICD code in Chile from 2010 to 2019. | | | | | | | | |  |
| --- | --- | --- | --- | --- | --- | --- | --- | --- | --- |
|  |  |  |  |  |  |  |  |  |  |
| **Variable** |  | **ICD-10 N18 codes for CKD** | | | | |  |  |  |
|  | **Total** | **N18.1** | **N18.2** | **N18.3** | **N18.4** | **N18.5** | **N18.6** | **N18.9** |  |
| **Number of hospitalizations** (%) | 99,816 (100) | 458 (0.5) | 190 (0.2) | 1,077 (1.1) | 2,694 (2.7) | 25,387 (25.4) | 0 (0) | 70,010 (70.1) |  |
| **Sex (%)** |  |  |  |  |  |  |  |  |  |
| Men | 52,583 (52.7) | 241 (52.6) | 94 (49.5) | 574 (53.3) | 1,369 (50.8) | 13,195 (52.0) | 0 (0) | 37,110 (53.0) |  |
| Women | 47,233 (47.3) | 217 (47.4) | 96 (50.5) | 503 (46.7) | 1,325 (49.2) | 12,192 (48.0) | 0 (0) | 32,900 (47.0) |  |
| **Age (mean, SD)** | 60.1 (18.7) | 58.9 (21.8) | 54.5 (23.8) | 64.9 (19.8) | 63.2 (18.1) | 58.5 (19.0) | - | 60.5 (18.6) |  |
| **Age group (%)** |  |  |  |  |  |  |  |  |  |
| 0 – 19 | 4,012 (4) | 44 (9.6) | 27 (14.2) | 48 (4.5) | 88 (3.3) | 1,317 (5.2) | 0 (0) | 2,488 (3.6) |  |
| 20 – 39 | 9,146 (9.2) | 31 (6.8) | 14 (7.4) | 60 (5.6) | 185 (6.9) | 2,436 (9.6) | 0 (0) | 6,420 (9.2) |  |
| 40 – 59 | 25,629 (25.7) | 103 (22.5) | 50 (26.3) | 196 (18.2) | 584 (21.7) | 7,026 (27.7) | 0 (0) | 17,670 (25.2) |  |
| 60 – 79 | 46,522 (46.6) | 208 (45.4) | 73 (38.4) | 505 (46.9) | 1,323 (49.1) | 11,521 (45.4) | 0 (0) | 32,892 (47.0) |  |
| 80+ | 14,507 (14.5) | 72 (15.7) | 26 (13.7) | 268 (24.9) | 514 (19.1) | 3,087 (12.2) | 0 (0) | 10,540 (15.1) |  |
| Length of stay (days) | 6 (1 - 946) | 4 (1 - 185) | 5 (1 - 141) | 5 (1 - 166) | 7 (1 - 93) | 7 (1 - 946) | - | 5 (1 - 760) |  |
| (median. range) |  |  |  |  |  |  |  |  |  |
| **In-hospital Mortality** (%) |  |  |  |  |  |  |  |  |  |
| Dead | 5,725 (5.7) | 20 (4.4) | 9 (4.7) | 34 (3.2) | 105 (3.9) | 1,487 (5.9) | 0 (0) | 4,070 (5.8) |  |
| Alive | 94,091 (94.3) | 438 (95.6) | 181 (95.3) | 1,043 (96.8) | 2,589 (96.1) | 23,900 (94.1) | 0 (0) | 65,940 (94.2) |  |
| **ICD-10 codes: N18.1** Chronic kidney disease, stage 1. **N18.2** Chronic kidney disease, stage 2. **N18.3** Chronic kidney disease, stage 3. **N18.4** Chronic kidney disease, stage 4. **N18.5** Chronic kidney disease, stage 5. **N18.6** End stage renal disease. **N18.9** Chronic kidney disease, unspecified | | | | | | | | |  |

| **Supplementary Table 3.** Annual Percent Change (APC) in Age-Adjusted Hospitalization Rates for Acute Kidney Injury by Region in Chile from 2010 to 2019. | | | | | | |  |
| --- | --- | --- | --- | --- | --- | --- | --- |
|  |  |  |  |  |  |  |  |
|  |  |  |  |  |  |  |  |
| Region | Time period | | Region and time period | APC (%) | CI 95% | |  |
|  | Lower EndPoint | Upper Endpoint |  |  | Lower CI | Upper CI |  |
| Arica and Parinacota | 2010 | 2019 | Arica and Parinacota Region  2010 - 2019 | 0.33 | -12.71 | 14.77 |  |
| Tarapacá | 2010 | 2017 | Tarapacá Region  2010 - 2017 | -5.10 | -36.75 | 44.01 |  |
| Tarapacá | 2017 | 2019 | Tarapacá Region  2017 - 2019 | 28.85 | -14.69 | 74.74 |  |
| Antofagasta | 2010 | 2014 | Antofagasta Region  2010 - 2014 | -8.24 | -22.57 | -0.76 |  |
| Antofagasta | 2014 | 2019 | Antofagasta Region  2014 - 2019 | 7.68 | 1.97 | 25.08 |  |
| Atacama | 2010 | 2019 | Atacama Region  2010 - 2019 | -2.69 | -8.58 | 3.35 |  |
| Coquimbo | 2010 | 2019 | Coquimbo Region  2010 - 2019 | 0.15 | -5.29 | 6.07 |  |
| Valparaíso | 2010 | 2019 | Valparaíso Region  2010 - 2019 | 7.21 | 5.20 | 9.54 |  |
| Metropolitana de Santiago | 2010 | 2014 | Metropolitan Region  2010 - 2014 | 4.32 | -7.45 | 10.30 |  |
| Metropolitana de Santiago | 2014 | 2019 | Metropolitan Region  2014 - 2019 | 14.83 | 11.40 | 23.82 |  |
| O'Higgins | 2010 | 2017 | O’Higgins Region  2010 - 2017 | 3.62 | -3.89 | 6.90 |  |
| O'Higgins | 2017 | 2019 | O’Higgins Region  2017 - 2019 | 29.83 | 13.05 | 44.35 |  |
| Maule | 2010 | 2012 | Maule Region  2010 - 2012 | -6.84 | -16.81 | 8.43 |  |
| Maule | 2012 | 2019 | Maule Region  2012 - 2019 | 13.50 | 11.35 | 20.99 |  |
| Ñuble | 2010 | 2019 | Ñuble Region  2010 - 2019 | 4.20 | -3.20 | 12.79 |  |
| Bio-Bío | 2010 | 2012 | Bío-bío Region  2010 - 2012 | 22.21 | 12.01 | 32.17 |  |
| Bio-Bío | 2012 | 2019 | Bío-bío Region  2012 - 2019 | 6.80 | 4.64 | 8.02 |  |
| La Araucanía | 2010 | 2019 | La Araucanía Region  2010 - 2019 | 15.30 | 9.93 | 23.64 |  |
| Los Ríos | 2010 | 2019 | Los Ríos Region  2010 - 2019 | 8.03 | -1.42 | 20.81 |  |
| Los Lagos | 2010 | 2013 | Los Lagos Region  2010 - 2013 | 28.97 | 19.31 | 48.59 |  |
| Los Lagos | 2013 | 2019 | Los Lagos Region  2013 - 2019 | 1.71 | -1.31 | 4.07 |  |
| Aysén | 2010 | 2019 | Aysén Region  2010 - 2019 | 19.99 | 13.43 | 31.85 |  |
| Magallanes | 2010 | 2013 | Magallanes Region  2010 - 2013 | 67.00 | 29.33 | 218.56 |  |
| Magallanes | 2013 | 2019 | Magallanes Region  2013 - 2019 | -0.50 | -13.57 | 7.09 |  |
| Nationwide | 2010 | 2019 | Nationwide  2010 - 2019 | 9.10 | 8.03 | 10.46 |  |
| Source: Based on data from the vital statistics system, hospital discharges, DEIS, MINSAL. | | | | | | |  |
| * Software ®JoinPoint. | | | | | | |  |

| **Supplementary Table 4.** Annual Percent Change (APC) in Age-Adjusted Hospitalization Rates for Chronic Kidney Disease, by Region in Chile from 2010 to 2019. | | | | | | | |  |
| --- | --- | --- | --- | --- | --- | --- | --- | --- |
|  |  |  |  |  |  |  |  |  |
|  |  |  |  |  |  |  |  |  |
| Region | Segment | Time period | | Region and time period | APC (%) | CI 95% | |  |
|  |  | Lower EndPoint | Upper Endpoint |  |  | Lower CI | Upper CI |  |
| Arica and Parinacota | 1 | 2010 | 2014 | Arica and Parinacota Region  2010 - 2014 | -8.40 | -22.28 | -0.33 |  |
| Arica and Parinacota | 2 | 2014 | 2019 | Arica and Parinacota Region  2014 - 2019 | 3.65 | -3.20 | 21.77 |  |
| Tarapacá | 1 | 2010 | 2019 | Tarapacá Region  2010 - 2019 | -13.55 | -19.09 | -9.01 |  |
| Antofagasta | 1 | 2010 | 2019 | Antofagasta Region  2010 - 2019 | -6.56 | -11.97 | -1.40 |  |
| Atacama | 1 | 2010 | 2013 | Atacama Region  2010 - 2013 | -21.82 | -41.29 | -5.38 |  |
| Atacama | 2 | 2013 | 2019 | Atacama Region  2013 - 2019 | 4.95 | -3.23 | 36.23 |  |
| Coquimbo | 1 | 2010 | 2019 | Coquimbo Region  2010 - 2019 | -4.32 | -7.04 | -1.66 |  |
| Valparaíso | 1 | 2010 | 2019 | Valparaíso Region  2010 - 2019 | -6.25 | -8.89 | -3.85 |  |
| Metropolitana de Santiago | 1 | 2010 | 2019 | Metropolitan Region  2010 - 2019 | -3.27 | -6.42 | -0.11 |  |
| O'Higgins | 1 | 2010 | 2016 | O’Higgins Region  2010 - 2016 | -5.96 | -8.77 | -4.00 |  |
| O'Higgins | 2 | 2016 | 2019 | O’Higgins Region 2016 - 2019 | 10.64 | 5.08 | 19.94 |  |
| Maule | 1 | 2010 | 2017 | Maule Region  2010 - 2017 | -7.97 | -13.63 | -6.04 |  |
| Maule | 2 | 2017 | 2019 | Maule Region  2017 - 2019 | 8.45 | -4.08 | 17.66 |  |
| Ñuble | 1 | 2010 | 2017 | Ñuble Region  2010 - 2017 | 3.07 | -2.97 | 39.34 |  |
| Ñuble | 2 | 2017 | 2019 | Ñuble Region  2017 - 2019 | -29.87 | -50.74 | -4.43 |  |
| Bio-Bío | 1 | 2010 | 2019 | Bío-bío Region  2010 - 2019 | -1.83 | -5.69 | 2.17 |  |
| La Araucanía | 1 | 2010 | 2019 | La Araucanía Region  2010 - 2019 | -3.22 | -9.29 | 3.29 |  |
| Los Ríos | 1 | 2010 | 2019 | Los Ríos Region  2010 - 2019 | -8.72 | -21.87 | 4.13 |  |
| Los Lagos | 1 | 2010 | 2017 | Los Lagos Region  2010 - 2017 | -1.19 | -3.62 | 10.66 |  |
| Los Lagos | 2 | 2017 | 2019 | Los Lagos Region  2017 - 2019 | -15.42 | -27.05 | -4.23 |  |
| Aysén | 1 | 2010 | 2016 | Aysén Region  2010 - 2016 | 7.41 | 1.71 | 34.79 |  |
| Aysén | 2 | 2016 | 2019 | Aysén Region  2016 - 2019 | -12.55 | -35.81 | -0.42 |  |
| Magallanes | 1 | 2010 | 2019 | Magallanes Region  2010 - 2019 | -2.79 | -5.31 | -0.28 |  |
| Nationwide | 1 | 2010 | 2019 | Nationwide  2010 - 2019 | -4.35 | -5.76 | -2.99 |  |
| Source: Based on data from the vital statistics system, hospital discharges, DEIS, MINSAL. | | | | | | | |  |
| Software ®JoinPoint. | | | | | | | |  |
